# Restoring Function After Severe Spinal Cord Injury Through BioLuminescent-OptoGenetics

**DOI:** 10.3389/fneur.2021.792643

**Published:** 2022-01-20

**Authors:** Eric D. Petersen, Erik D. Sharkey, Akash Pal, Lateef O. Shafau, Jessica Zenchak-Petersen, Alex J. Peña, Anu Aggarwal, Mansi Prakash, Ute Hochgeschwender

**Affiliations:** ^1^Program in Neuroscience, Central Michigan University, Mount Pleasant, MI, United States; ^2^College of Medicine, Central Michigan University, Mount Pleasant, MI, United States; ^3^Electrical and Computer Engineering, University of Illinois Urbana Champaign, Urbana, IL, United States

**Keywords:** optogenetic, bioluminescence, spinal cord injured (SCI), stimulation, chemogenetic

## Abstract

The ability to manipulate specific neuronal populations of the spinal cord following spinal cord injury (SCI) could prove highly beneficial for rehabilitation in patients through maintaining and strengthening still existing neuronal connections and/or facilitating the formation of new connections. A non-invasive and highly specific approach to neuronal stimulation is bioluminescent-optogenetics (BL-OG), where genetically expressed light emitting luciferases are tethered to light sensitive channelrhodopsins (luminopsins, LMO); neurons are activated by the addition of the luciferase substrate coelenterazine (CTZ). This approach utilizes ion channels for current conduction while activating the channels through the application of a small chemical compound, thus allowing non-invasive stimulation and recruitment of all targeted neurons. Rats were transduced in the lumbar spinal cord with AAV2/9 to express the excitatory LMO3 under control of a pan-neuronal or motor neuron-specific promoter. A day after contusion injury of the thoracic spine, rats received either CTZ or vehicle every other day for 2 weeks. Activation of either neuron population below the level of injury significantly improved locomotor recovery lasting beyond the treatment window. Utilizing histological and gene expression methods we identified neuronal plasticity as a likely mechanism underlying the functional recovery. These findings provide a foundation for a rational approach to spinal cord injury rehabilitation, thereby advancing approaches for functional recovery after SCI.

**Summary:**

Bioluminescent optogenetic activation of spinal neurons results in accelerated and enhanced locomotor recovery after spinal cord injury in rats.

## Introduction

The manipulation of specific neuronal populations of the spinal cord following spinal cord injury (SCI) could prove highly beneficial for rehabilitation in patients. This could work by maintaining and strengthening existing neuronal connections and/or facilitating neuronal growth and the formation of new synapses in a controlled, activity dependent manner. Stimulation of circuits in the spinal cord would ideally be highly cell type specific and non-invasive. Electrical stimulation presents a straight-forward means to activate neurons of the spinal cord and although having clinical promise, this approach has several limitations (1). Electrical stimulation excites all cells within the electrode vicinity including non-neuronal cells, potentially diluting, or negating the effect of targeted stimulation of specific beneficial cell types. Some types of electrical stimulation also result in rapid muscle fatigue by preferentially recruiting large, rapidly adapting motor units, limiting the on-time for stimulation (2, 3). Further, approaches to electrical stimulation require a chronic implant near the spinal cord, potentially increasing risk to the patient.

Optogenetics is a promising method for stimulating neurons of the spinal cord and overcomes some of the problems with electrical stimulation, allowing activation of specific channels or effectors that can be targeted to discrete, genetically unique neural sub-populations or glia. This allows treatment approaches to be tailored in highly specific and diverse ways by taking advantage of the plethora of genetic targeting strategies that are currently available and rapidly evolving. However, the need for invasive chronic optical fiber implants connected to an external light source or implanted LED modules pose problems for long-term treatment when applied to the spinal cord. Furthermore, light from an external source is limited in its ability to penetrate neural tissue at safe power levels. Yi and colleagues reported successful optic stimulation at a depth of nearly 300 μm below the dorsal surface of the spinal cord, successfully stimulating microglia of the dorsal lamina to induce chronic pain in mice (4). Others have successfully stimulated more ventral populations in rats (depth of ~1 mm), however requiring 40 to 50 mW/mm^2^ for ChR2 and Chronos, respectively (5). Although successful at activating neurons well-below the surface of the cord this level of radiance is not feasible for therapeutic applications. For example as little as 3 mW/mm^2^ can alter neuronal activity, 7 mW/mm^2^ can have measurable behavioral effects. These relatively low levels of light that are routinely used for optogenetic manipulations are also sufficient to cause microglial activation and tissue damage and heating (6–10). Taken together it is clear that alternative methods for neuronal stimulation that do not require external sources of illumination need to be explored as options for SCI treatment especially considering that the human spinal cord is over a centimeter thick.

BioLuminescent-OptoGenetics (BL-OG) is a recently developed approach that has the potential to overcome the barriers to clinical success presented by traditional optogenetic approaches for rehabilitation following SCI. BL-OG uses powerful optogenetic elements that do not require an external implant, but instead uses light generated internally by tethering bioluminescent luciferases to light sensitive channelrhodopsins, luminopsins (LMO). The bioluminescent light is produced by the breakdown of a specific enzymatic substrate, in this case coelenterazine (CTZ). Stimulation only occurs when the CTZ is injected, producing bioluminescent light through catalysis by the luciferase, resulting in the activation of the opsin. Different from other opto- and chemogenetic approaches BL-OG utilizes ion channels rather than GPCRs for current conduction while activating the channels through the application of a chemical compound, thus allowing non-invasive stimulation and recruitment of all targeted actuators as opposed to only those that can be reached by light from a physical source (11–17). Here we use LMO3 which consists of slow burn *Gaussia* luciferase fused to *Volvox* channelrhodopsin 1. This system has been demonstrated to consistently activate expressing neuronal cells with little to no off target effects caused by the substrate and bioluminescent reaction that takes place (18–22). Moreover, bioluminescence is light emitted without heat (“cold light”) and thus does not approach the damaging levels encountered for traditional optogenetics (23, 24). Utilizing LMOs for neural stimulation in the spinal cord presents an innovative approach for activating neurons that could likely be therapeutically beneficial to recovery following SCI that was not previously possible with other approaches.

Here we sought to determine if genetically targeted BL-OG stimulation restricted to neurons of the lumbar spinal cord would be beneficial for locomotor recovery following experimental spinal cord injury.

## Results

### Bioluminescent Optogenetic Stimulation of Spinal Neurons

**Figure 1:**
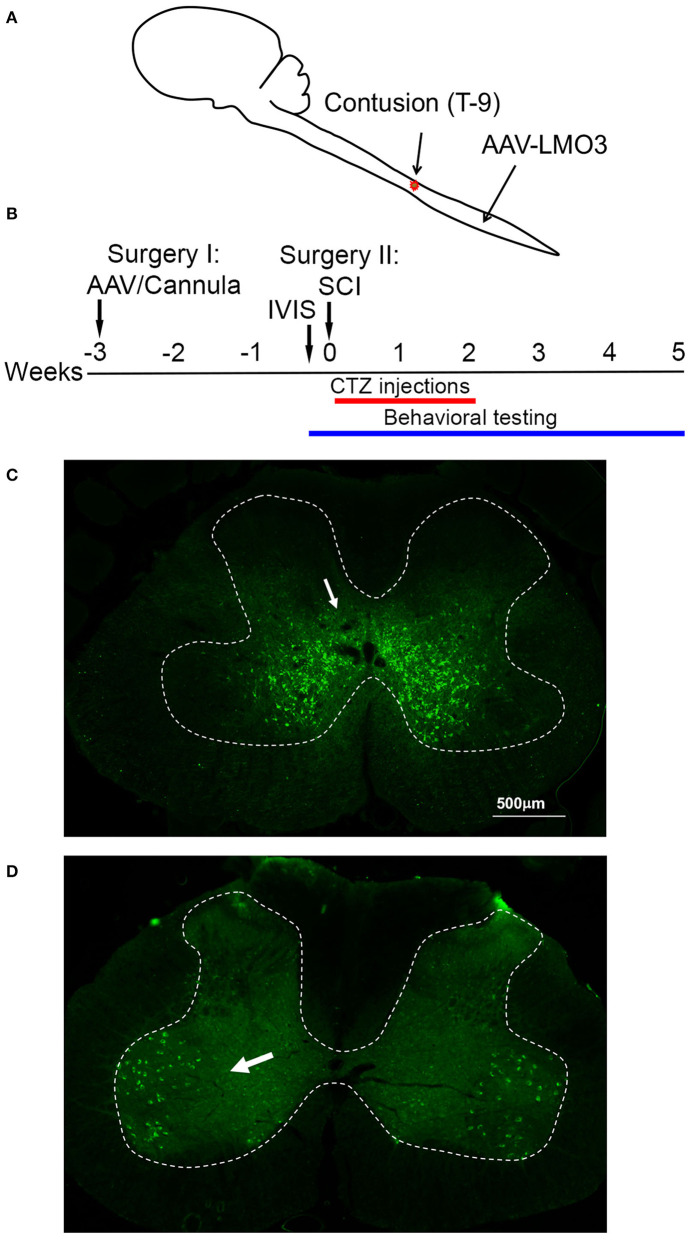
Spinal cord injury model. **(A)** Schematic of the experimental model with viral injection for BL-OG stimulation in the lumbar enlargement and contusion injury in the thoracic region. **(B)** Timeline of experimental procedures with the first surgery for lateral ventricle cannula placement and virus injection 3 weeks prior to injury. **(C)** Expression of AAV 2/9 hSyn-LMO3 in the lumbar spinal cord (arrow pointing to expressing interneurons). The highest levels of expression are restricted to interneuron populations in lamina IV-VIII and X with some expression more dorsal and in lamina IX. **(D)** Expression of AAV 2/9 Hb9-LMO3 in the lumbar spinal cord (arrow pointing to expressing motor neurons). The Hb9 promoter restricts expression to motor neurons in lamina IX. Some low level of expression does occur throughout other laminae of the cord.

To assess the possibility of BL-OG stimulation to re-engage neurons below the site of a spinal cord injury we transduced neurons of the lumbar enlargement with AAV vectors to express LMO3 (Figure 1A). At the time of AAV injection, we also implanted a lateral ventricle cannula for easy and controlled application of the luciferase substrate CTZ (Supplementary Figure 1). Contusion injury of the thoracic spinal cord was carried out 3 weeks later, followed by BL-OG stimulation and testing of locomotor behavior (Figure 1B). LMO3 expression was under control of a pan-neuronal human synapsin promoter (hSyn) or a motoneuron-specific Homeobox 9 promoter (Hb9). Expression under the hSyn promoter was consistently concentrated to neurons located within laminae IV-VIII and X, with some expression in lamina IX (Figure 1C). The Hb9 promoter successfully restricted expression almost exclusively to motor neurons in lamina IX (Figure 1D), with some interneurons also expressing the construct, which is consistent with previous reports (25–32). In the rostral—caudal dimension LMO3 expression with both promoters was observed throughout the majority of the lumbar enlargement, with the highest levels of expression closest to the injection site.

**Figure 2:**
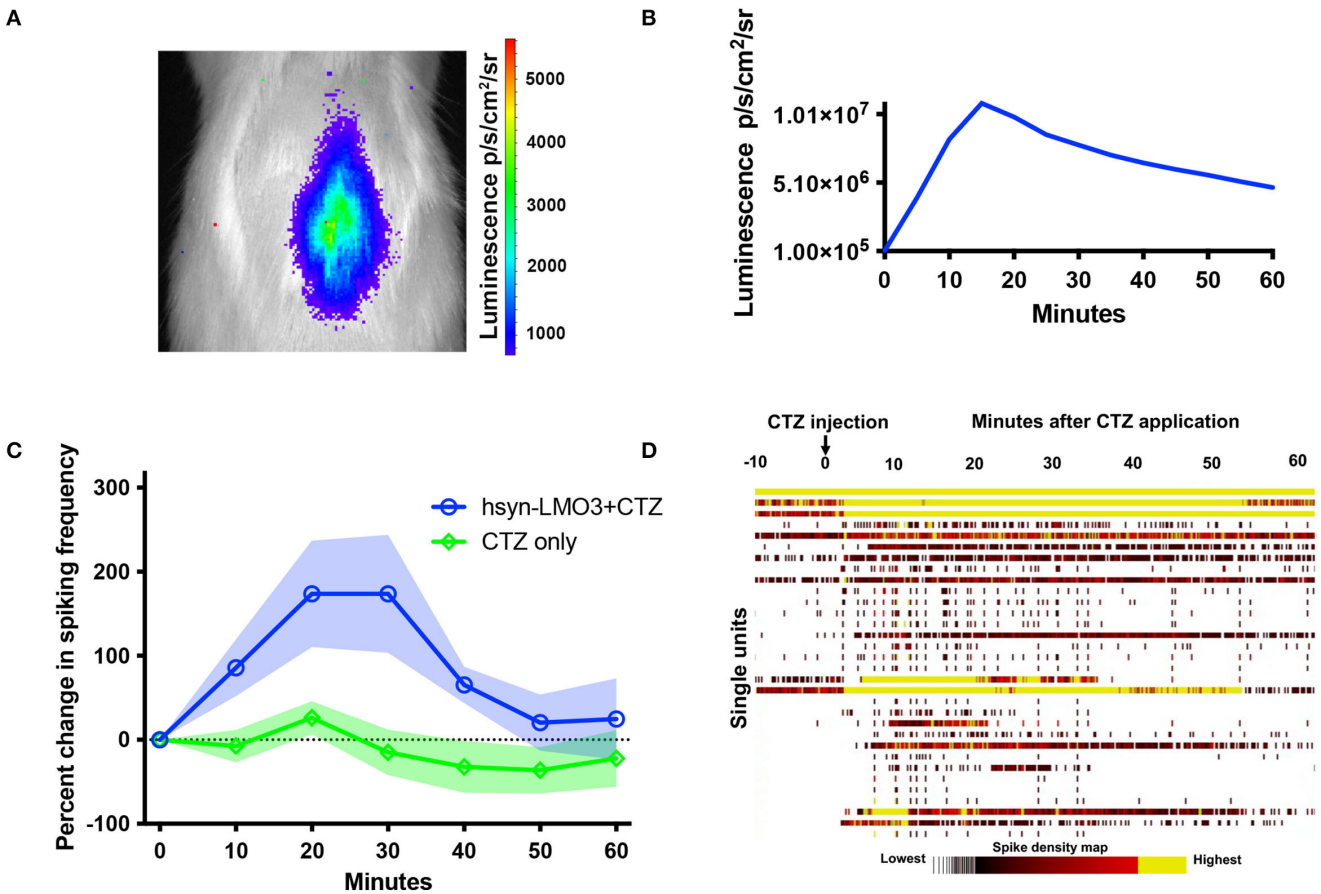
Bioluminescent optogenetic stimulation of spinal cord neurons. **(A)** Example of *in vivo* bioluminescent imaging of a rat expressing LMO3 in the lumbar spinal cord following CTZ infusion through the lateral ventricle. Luminescence (pseudocolored) is localized over the lumbar region of the cord. **(B)** A representative trace of luminescence over time following CTZ infusion in the lateral ventricle. **(C)** Single unit electrophysiological response in the lumbar spinal cord of rats expressing AAV 2/9 hSyn-LMO3 compared to non-expressing animals when CTZ is infused through the lateral ventricle. Similar to luminescence over time, activity increases and peaks between 10 and 30 min following CTZ infusion. *n* = 7 for LMO3 expressing and *n* = 4 for non-expressing animals that received CTZ. Shading = SEM. **(D)** Raster plot of the response to CTZ in an LMO3 expressing rat.

To insure that viral transductions resulted in LMO3 expression at levels sufficient for neuronal activation and in the intended anatomical region, we took advantage of the unique feature of LMOs allowing for *in vivo* bioluminescence imaging to report expression of the protein and confirm successful administration of the substrate. Bioluminescence was detected over the lumbar region of the spinal cord in rats transduced with LMO3 (Figure 2A). Light intensities over time consistently peaked between 10 and 30 min post CTZ application and decayed over the next hour (Figure 2B). Utilizing *in vivo* bioluminescent imaging not only allowed us to confirm LMO3 expression, we were also able to verify proper cannula function. As we performed *in vivo* bioluminescent imaging prior to the contusion injury on each animal, we avoided continuing with animals that had insufficient signal, due to very low or no expression or due to a non-functional cannula.

Next we determined the effect of LMO stimulation on activity of spinal neurons by recording from the AAV injection site with multichannel electrodes when CTZ is infused through the ventricle. We confirmed that increases in neuronal activity within the lumbar spinal cord followed a similar timeline as observed with *in vivo* bioluminescent imaging (Figures 2C,D). When testing the electrophysiological effect of CTZ on naïve rats under the same recording conditions, we did not find any change in activity over baseline spiking rates (Figure 2D).

### Bioluminescent Optogenetics Results in Accelerated and Enhanced Locomotor Recovery After SCI

**Figure 3:**
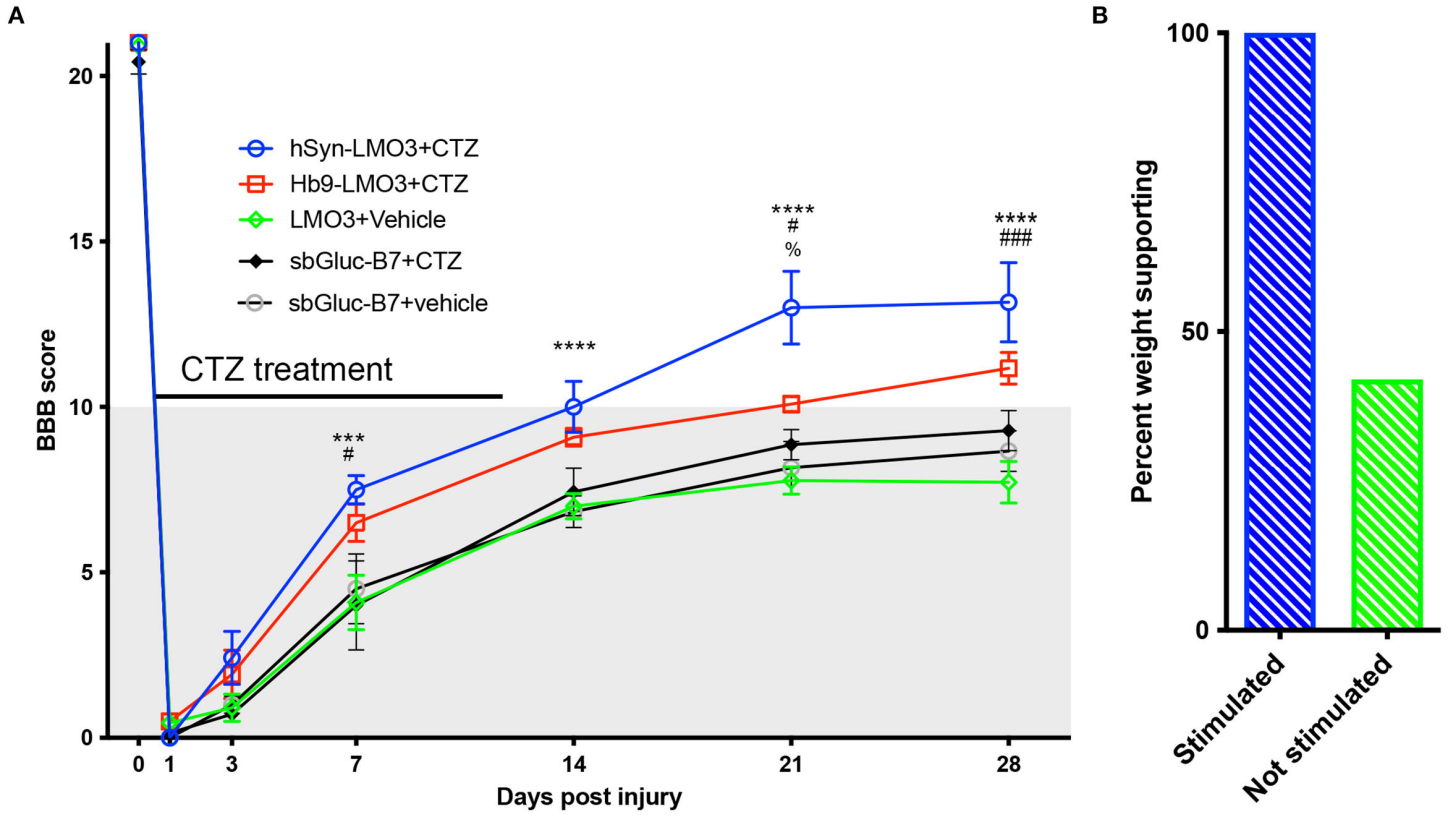
Accelerated and enhanced locomotor recovery with BL-OG. **(A)** BBB locomotor scores following injury and treatment for animals expressing LMO3 in neurons (hSyn) or specifically in motor neurons (Hb9) or expressing just the luciferase in neurons (sbGLuc-B7). Animals received either CTZ or vehicle following injury. Those which received neural stimulation regardless of neuronal subpopulation (hSyn-LMO3 + CTZ, Hb9-LMO3 + CTZ) recovered at a faster rate, to a greater extent, and maintained their status following the treatment period compared to the non-stimulated vehicle treated group (LMO3+veh) as well as the groups expressing only the luciferase. For Bonferroni *post-hoc*: hSyn-LMO3+CTZ vs vehicle *p < 0.05, **p < 0.01, ***p < 0.001, ****p < 0.0001; Hb9-LMO3 + CTZ vs. vehicle ^#^*p* < 0.05, ^*##*^*p* < 0.01, ^*###*^*p* < 0.001; hSyn-LMO3 + CTZ vs. Hb9-LMO3 + CTZ; %*p* < 0.05. *n* = 6 for hSyn-LMO3 + CTZ, *n* = 6 for Hb9-LMO3 + CTZ, *n* = 11 for vehicle treated animals. Animals expressing the luciferase only were not significantly different from the vehicle treated controls. **(B)** Comparison of the percentage of weight supporting animals at the endpoint (28 days) for those that received BL-OG stimulation (hSyn-LMO3 + CTZ, Hb9-LMO3 + CTZ) compared to all three control groups (LMO3 + veh, sbGluc-B7 + CTZ, sbGluc-B7 + veh).

All animals expressing LMO3 in the lumbar spinal cord had Basso, Beattie, and Bresnahan (BBB) ratings of 21 (perfect gait) before undergoing surgery for spinal cord injury. After thoracic contusion injury rats were randomly assigned to two groups with one group receiving CTZ and the other group receiving vehicle via ventricular infusion. Applications were delivered every other day for 14 days, starting the day after SCI surgery. SCI rats that received CTZ mediated neural stimulation showed a significant improvement in locomotor scores which persisted even after the treatment period (Figure 3A). Animals that received stimulation via hSyn-LMO3 (*n* = 6) had a final mean BBB score of 13.2, representing animals with frequent to consistent weight supported plantar steps and frequent front limb-hind limb coordination. Animals that received stimulation via Hb9-LMO3 (*n* = 6) had a mean BBB score of 11.2, representing animals able to take frequent to consistent weight supported steps. Those treated with the vehicle (*n* = 11) had a final mean BBB score of 7.7, representing animals that are able to move both hindlimbs in a sweeping motion without any weight support. Animals receiving BL-OG mediated stimulation regardless of the neuronal population targeted improved at a faster rate than vehicle treated controls, with significantly better locomotor scores from days 7–28 post injury. For locomotor recovery scores, there was a significant main effect for treatment [*F*_(4, 31)_ = 10.31, *p* = 2.0 × 10^−5^] and a significant main effect for time [*F*_(6, 186)_ = 843.46, *p* < 1.0 × 10^−15^]. There was also a significant interaction effect for treatment by time point [*F*_(24, 186)_ = 3.29, *p* = 3.0 × 10^−6^]. We also sought to determine if locomotor recovery could be explained by off target effects of bioluminescence. For this, we tested whether bioluminescence produced by the luciferase sbGluc and substrate CTZ without an optogenetic channel present could impact recovery. We used the construct hSyn-sbGLuc-B7-EYFP where B7 is a transmembrane domain replacing the optogenetic channel so the luciferase is extracellular and tethered to the cell membrane as in the LMO constructs. This tested the potential effects of bioluminescence, CTZ, and all breakdown products from the chemical reaction. Following the same injury and treatment protocol used for LMO expressing animals, we found no difference at any time point between sbGLuc-B7-EYFP expressing animals receiving CTZ and vehicle (Figure 3A). Those treated with hSyn-LMO3 + CTZ were significantly different from all control groups from day 7 post injury onward. HSyn-LMO3 + CTZ differed significantly at day 21 post injury from Hb9-LMO3 + CTZ. Hb9-LMO3 + CTZ differed significantly from LMO3 + vehicle at days 7, 21, and 28 and from SbGluc-B7 + CTZ at day 7. None of the three control groups tested differed significantly from each other at any timepoint.

At the experimental endpoint, 100% of rats that received stimulation were able to take weight bearing steps (BBB of 10 or higher) while only 35% of vehicle treated animals were able to take weight bearing steps (Figure 3B). We also found animals that received stimulation tended to regain bladder control sooner than the vehicle treated group, however this difference was not significant but could warrant further study (Supplementary Figure 2).

### Bioluminescent Optogenetics Effects Recovery After SCI Through Increasing Neuronal Plasticity

**Figure 4:**
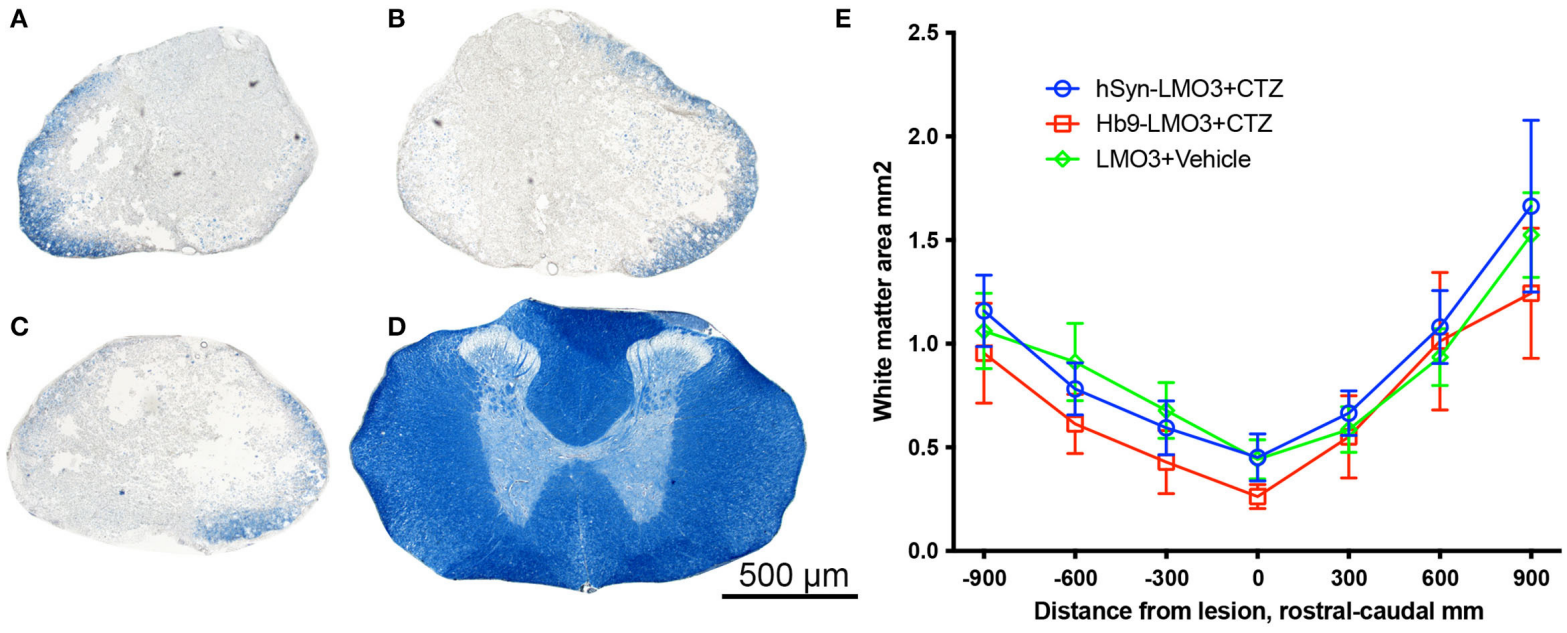
No sparing of white matter at the injury site. **(A–D)** Cross sections of spinal cords stained with eriochrome cyanin which stains white matter blue. **(A)** hSyn-LMO3 + CTZ; **(B)** Hb9-LMO3 + CTZ; **(C)** Vehicle treated; **(D)** Example of the white matter present in the same region of the spinal cord in a non-injured rat. **(E)** Comparison of the cross sectional area of spared white matter following injury and treatment. There were no differences in the amount of degeneration that occurred as a result of the contusion injury with or without neural stimulation.

The positive effect of post-injury engagement of neurons could be based on different mechanisms or a combination thereof. Since the treatment used was an early intervention, it could have influenced the extent of degeneration at the lesion site in a variety of ways. To assess sparing of myelinated white matter at the site of injury, eriochrome cyanin staining was used. We found no differences between either stimulation treatment condition and the vehicle treatment in the cross sectional area of preserved white matter at the lesion epicenter, excluding a major effect on alleviation of tissue degeneration (Figure 4).

Based on these results, we performed an additional experiment to determine if BL-OG stimulation is able to influence neuronal plasticity under the conditions used in this study. For this, we performed qRT-PCR with tissue from the region stimulated (lumbar) at 8 days post injury, during the treatment window. The experiment was carried out as described above with fresh tissue collection on day 8 post injury, when rats had received four CTZ applications. We chose this time point as it coincides with the first significant increase in locomotor recovery of CTZ treated vs. vehicle treated animals. When assessing gene expression at this time point we found animals that received stimulation had higher levels of all of the markers for neuronal plasticity that we tested for. These markers signify growth and remodeling in different regions of neurons: GAP-43, MAP2, PSD-95, and NMDAR2d (Table 1) which represent axonal growth, dendritic growth and synaptic remodeling, respectively. The upregulation of these genes further support the conclusion that improved recovery from injury with optogenetic stimulation is mediated by inducing neuronal plasticity. We also tested expression levels for genes associated with inflammation and apoptosis to determine if either of these have a role in promoting recovery. We did not find consistent trends for either inflammation or apoptotic markers when comparing animals treated with CTZ vs. vehicle.

**Table 1:** Gene expression levels after injury and treatment.

**Marker**	**Function**	**Fold of sham (SE)**	**CTZ percent of Veh**	**ANOVA**	**Bonferroni *post-hoc***
GAP-43	Axon growth	CTZ: 6.49 (1.49) Veh: 2.99 (1.65) Sham: 1.00	217%	*p* = 0.034 *F* = 4.52314	CTZ–veh: *p* = 0.248 CTZ–sham: *p* = 0.035 Veh–sham: *p* = 0.909
MAP2	Dendrite growth	CTZ: 12.56 (3.49) Veh: 3.65 (1.55) Sham: 1.00	344%	*p* = 0.014 *F* = 6.40513	CTZ–veh: *p* = 0.059 CTZ–sham: *p* = 0.020 Veh–sham: *p* = 1.000
PSD-95	Post synaptic structure	CTZ: 5.07 (1.43) Veh: 1.53 (0.77) Sham: 1.00	331%	*p* = 0.037 *F* = 4.52013	CTZ–veh: *p* = 0.093 CTZ–sham: *p* = 0.065 Veh–sham: *p* = 1.000
NMDAR2d	Post synaptic receptor	CTZ: 6.85 (2.30) Veh: 1.79 (0.75) Sham: 1.00	383%	*p* = 0.023 *F* = 5.27114	CTZ–veh: *p* = 0.071 CTZ–sham: *p* = 0.031 Veh–sham: *p* = 1.000
BDNF	Neurotrophic factor	CTZ: 0.56 (0.14) Veh: 0.75 (0.09) Sham: 1.00	75%	*p* = 0.001 *F* = 13.35214	CTZ–veh: *p* = 0.122 CTZ–sham: *p* = 0.001 Veh–sham: *p* = 0.043
VEGF	Neurotrophic factor	CTZ: 17.33 (4.33) Veh: 3.55 (1.90) Sham: 1.00	488%	*p* = 0.029 *F* = 4.96413	CTZ–veh: *p* = 0.156 CTZ–sham: *p* = 0.034 Veh–sham: *p* = 1.000
iNOS	M1 microglia/macrophage	CTZ: 8.98 (1.22) Veh: 5.70 (2.45) Sham: 1.00	157%	*p* = 0.028 *F* = 5.00513	CTZ–veh: *p* = 0.586 CTZ–sham: *p* = 0.027 Veh–sham: *p* = 0.269
Arginase1	M2 microglia/macrophage	CTZ: 11.48 (5.62) Veh: 7.24 (6.37) Sham: 1.00	158%	*p* = 0.118 *F* = 2.66212	CTZ–veh: *p* = 0.225 CTZ–sham: *p* = 0.246 Veh–sham: *p* = 1.000
Caspase 3	Apoptotic	CTZ: 1.31 (0.29) Veh: 1.26 (0.89) Sham: 1.00	104%	*p* = 0.487 *F* = 0.76813	CTZ–veh: *p* = 0.950 CTZ–sham: *p* = 0.914 Veh–sham: *p* = 1.000
Bcl-2	Anti-apoptotic	CTZ: 1.85 (0.48) Veh: 0.60 (0.36) Sham: 1.00	308%	*p* = 0.264 *F* = 1.50713	CTZ–veh: *p* = 0.349 CTZ–sham: *p* = .0889 Veh–sham: *p* = 1.000

*Comparison of relative mRNA levels for various biomarkers in the lumber spinal cord following injury and subsequent treatment. All levels are expressed as fold change over non injured sham animals. N = 5 for all groups except for MAP2, PSD-95,VEGF, iNOS, Caspase 3, and BCL-1, where one animal from the sham group did not have detectable levels of the gene of interest and was excluded, leaving N = 4 animals per group. For Arginase 1, one animal from sham and one from vehicle did not have a detectable level of the gene of interest and was excluded, leaving N = 4 animals for each of these 2 groups*.

## Discussion

The majority of spinal cord injuries are contusion injuries that leave behind areas of intact neural tissue below the site of injury. Neurons from those areas often maintain intact connections, even across the site of injury, yet patients are paralyzed. To explore if functionally ineffective or dormant populations of neurons below the injury could be re-engaged to activate spinal circuitry resulting in improved functional output, we stimulated neurons below the site of a severe contusion injury for 2 weeks using a bioluminescent optogenetics approach. We found that stimulating neurons with this approach improves the rate and extent of locomotor recovery following injury.

Optogenetic stimulation following spinal cord injury has been tested in a mouse model of cervical SCI previously and was found capable of improving breathing following treatment (33). However, the practical challenges of light delivery to often centrally located spinal cord target populations has hampered application of this approach to SCI in the case of activating the motor units associated with the diaphragm. Optogenetic stimulation has also been shown to successfully alleviate tail muscle spasms in a mouse sacral SCI model via activation of inhibitory interneurons, although the downfall of this approach is that activation of the motor units is completely suppressed, thus a need for further refinement (34). In principle, chemogenetic approaches are ideal for manipulation of spinal cord neurons as they require application of a chemical to activate genetically targeted neurons without requirements for hardware. In one study, Chen et al. restored stepping ability in mice with staggered bilateral hemisections by administering a KCC2 agonist. The same result was achieved by selective expression of hyperpolarizing DREADDs (hM4Di) in inhibitory interneurons between and around the staggered spinal lesions (35). However, chemogenetic approaches to more clinically relevant SCI injury models are lacking and the chemogenetic ligands often have off target effects (36). Using a rat spinal cord contusion model we explored BL-OG, an opto-chemogenetic approach that takes advantage of using ion channels for current conduction rather than GPCRs, thus making it independent of requirements for expression of specific GPCR coupled pathways.

Using BL-OG, we found that stimulating either primarily interneurons or primarily motor neurons resulted in significant functional improvements at faster rates and to a greater extent in stimulated animals compared to control animals. Of note is that for the motor neuron stimulation paradigm (Hb9-LMO3), there are relatively few neurons that express the LMO construct compared to the pan neuronal paradigm (hSyn-LMO3) where all neurons can express the construct, yet a very similar end result was achieved. Our findings of improvement regardless of the neuronal population that was stimulated is different from those in a study in mice where directly reducing the excitability of inhibitory Vgat interneurons, but not directly increasing the excitability of Vglut excitatory interneurons resulted in improvement after bilateral hemisections (35). Putting the differences in SCI model, species, and neural populations targeted aside, the results from both studies strengthen the concept that selective stimulation of dormant neurons promotes recovery. It will be of great interest to determine if targeting BL-OG stimulation to other genetically distinct neural subpopulations could further improve outcomes following SCI. For example, there exist a variety of genetically identifiable interneuron subtypes that make up mammalian central pattern generators (CPGs) in the spinal cord, including some which remain to be fully characterized that will likely prove useful targets to promote recovery following SCI although a means to target these populations in a clinical setting have yet to be developed (37).

Initially, the expectation was that CTZ presentation in hSyn-LMO3 animals would cause a transient disruption in gait coordination due to the simultaneous activation of counteracting CPG interneurons and both excitatory and inhibitory populations. In contrast, the Hb9-LMO3 animals were expected to be able to maintain coordination during the stimulation and improve to a greater extent than the hSyn-LMO3 animals. The basis for this prediction was that although mammalian spinal motor neurons are generally considered to be the output elements of the spinal cord, stimulation of motor neurons can induce episodes of locomotor activity driven by the lumbar CPGs (38). Excitation of motor neurons in the spinal cord are known to reciprocally activate ventrolaterally located spinal networks through the actions of excitatory interneurons (39). Based on this, we expected stimulation of only motor neurons to be beneficial in maintaining the precise timing of alternating CPG interneuron pools necessary to carry out coordinated locomotion (37). One explanation for not observing notable differences in this regard is that the animals were receiving stimulation at an early time during recovery when they were not walking yet with any degree of coordination. Considerations about neural stimulation not interfering with CPG patterns might be more significant when this approach is used for treatment of less severe injuries, at later time points, for longer periods of time, and in conjunction with physical therapy. Another possibility is that regardless of what neuronal subpopulation was stimulated, the optogenetic stimulation could prime neural networks of the lumbar region to undergo strengthening of synaptic connections with supraspinal projections and within the network to develop more effective locomotor networks that are able to compensate following injury. It is also possible that these changes are largely driven by subthreshold optogenetic stimulation. In the case of BL-OG, there is a rising phase and prolonged falling phase in light production and neural activity as the CTZ concentration diffuses to the lumbar region of the cord and as the substrate is slowly used up and cleared away. This creates a much larger window where subthreshold actions of the LMO may be able to influence network dynamics. It remains to be seen if other LMO variants, such as the highly sensitive step function LMOs, are more effective for treating neuronal injuries; alternatively, it is possible that overstimulation could be less beneficial or even detrimental (13, 40). These interesting results can be built upon in future studies to determine if targeting specific subsets of interneurons can be more beneficial (41).

The hypothesis that BL-OG stimulation of locomotor networks can induce remodeling at the neuronal and synaptic level is supported by our results. We did not find an effect on sparing of white matter that could have had a large impact on behavioral outcomes. This led us to directly test the effect of LMO stimulation on markers for plasticity. In RT-PCR experiments we found all four neuronal plasticity markers encompassing axon, dendrite, and synaptic remodeling to be expressed at higher levels after injury but even more so as a result of treatment. We also tested a variety of other biomarkers to determine if inflammation was affected and did not find evidence of altered inflammatory state as a result of stimulation. All together, we believe that BL-OG induced recovery following SCI is largely mediated by optogenetically induced neuronal plasticity and potentially maintenance of neural networks. We expect that a mechanism for recovery could be elucidated further by employing more advanced single cell RNAseq techniques in future studies to identify potential spinal neuron populations that mediate recovery and are ideal targets for selective BL-OG stimulation (42). In a previous study of optogenetic stimulation of cervical SCI, beneficial effects were largely enabled by neuronal plasticity although this was an acute study (33). Consistent with these findings, optogenetics has previously been demonstrated to induce neuronal plasticity *in vitro* and *in vivo*, and to promote recovery following different types of neural trauma (33, 43–45). Most recently, BL-OG stimulation has been used to restore function following stroke, where the benefits of stimulation were also found to be a result of optogenetically induced neuronal plasticity (19). It may also be possible that BL-OG stimulation influences plasticity in neurons even if they are not directly recruited by the addition of CTZ but potentially by bringing their membrane potential closer to threshold, thus making them more excitable.

Our results are highly encouraging in the context of clinical translatability. Viral vectors for gene delivery are increasingly finding their way into the clinics as well as clinical trials using optogenetics. Coelenterazine has been used without detriment in animal imaging studies for decades. The specific route of application in our study, lateral ventricle infusion, was chosen based on considerations of practicability in rats and expense for ip injections in rats, although with improved LMOs CTZ dosages will be able to be decreased by over ten fold making ip delivery in rats feasible. For human application, alternate routes would apply (intravenous, intranasal, oral). We report here on our initial, limited study that needs to be followed up to address several critical issues. For example, although our contusion injury is severe, we don't know if we reached the limit of effectiveness with our stimulation treatment. Going in the other direction, in cases of less severe contusions and expected higher numbers of preserved intact neurons below the site of injury, it is possible that detrimental effects such as muscle spasticity start arising with over stimulation. While we did not observe any behavior in neurally stimulated animals that would suggest their experience of pain, any potential effects on pain will have to be assed in depth in future experiments. Furthermore, it will be of clinical importance to try the BL-OG stimulation approach at later time points after the occurrence of the injury. Lastly, we might see synergistic effects when combining BL-OG stimulation with physical exercises.

From a translational perspective we expect that our results can be built upon in the future to develop improved approaches to treating SCI that leverage the capacity of optogenetic stimulation for induction of plasticity for successful treatment of patients. Given the observation in our study that animals that received stimulation tended to regain bladder function sooner, it will be promising to explore this approach as a means to improve bladder control by targeting the stimulation purposely to the nuclei of the cord that are responsible for bladder control. This would be a major quality of life improvement for patients with SCI.

## Materials and Methods

### Animals

Adult female Sprague Dawley rats, 4–6 months of age, bred on site, weighing 280–350 g were used. All experimental procedures were performed in accordance with guidelines from the NIH and were approved by the Central Michigan University Institutional Animal Care and Use Committee (IACUC). Animals were kept in 12 h light/dark cycle rooms and fed *ad libitum*.

### Plasmids and Virus

LMO3, the third generation of excitatory LMOs, was expressed in neurons of the lumbar spinal cord utilizing an adeno-associated virus serotype 2/9. LMO3 consists of slow burn *Gaussia* luciferase fused to *Volvox* channel rhodopsin 1, with a yellow fluorescent protein tag and was expressed under the human synapsin (hSyn) promoter, which restricts gene expression to only neurons or under the rat Homobox 9 (Hb9) promoter, which restricts expression to motor neurons (25–29). A rat version of the promoter described in references (25–29), which is 99% similar to the mouse version was synthesized by Genscript and cloned into the pAAV-hSyn-LMO3 plasmid to replace the hSyn promoter, creating pAAV-Hb9-LMO3 using standard restriction cloning techniques (Addgene plasmid: 114103). The B7 transmembrane sequence from the mouse CD80 antigen was cloned into the AAV vector to replace the optogenetic channel (46). Plasmids were confirmed by sequencing. High titer stocks of hSyn-LMO3 virus were made by ViroVek. The other two viruses were made in-house using previously described methods for triple plasmid transfection in HEK293FT cells to encapsulate the constructs in a pseudotyped 2/9 capsid (14).

### Surgery

All surgeries were conducted under aseptic conditions.

#### Lateral Ventricle Cannulation

The lateral ventricle cannula consists of an infusion cannula (3280PM/SPC cut 4 mm below pedestal, Plastics One) to access the ventricle that is externalized through a PinPort (VABR1B/22, Instech Labs) that allows repeated aseptic access. The two parts are connected by 2.0 cm of 22G polyurethane tubing (VAHBPU-T22, Instech labs) (Supplementary Figure 1). For placement, an incision was made to expose the skull, periosteum removed, and bone dried thoroughly. A burr hole was drilled at −1.0 mm from bregma, 1.5 mm right of the midline for insertion of the cannula (47). Three machine screws (00–96 c 3/32 Plastics One) were inserted into hand drilled holes (D69, Plastics One) 0.742 mm forward, behind, and to the left of where the infusion cannula would be placed. The infusion cannula was lowered 4 mm below the skull and secured to the skull and screws by dental acrylic. The port was externalized though the skin on the neck and sutured tightly around the base with a 4-0 silk suture, and incisions closed with staples. Cannulas were kept clear from obstruction by infusing saline twice a week prior to the second surgery.

#### Viral Injections

During the same surgery, animals received viral injections in the lumbar spinal cord. The spinal cord was exposed by making an incision over the T-13/L1 vertebra and the soft tissue between the two vertebra was cleared to expose a minimal amount of the cord. The spinal column was stabilized using vertebral clamps. The virus was infused using a 10 μL World Precision Instruments syringe with 35G beveled needle. The virus was injected 0.5 mm lateral to the midline and 1.5 mm ventral to the surface at the following volumes per side: 2.5 μL at 1 × 10^13^ copies/mL for hSyn-LMO3, 6 μL at 5 × 10^12^ copies/mL for Hb9-LMO3, and 2.5 μL at 3 × 10^12^ for hSyn-sbGluc-B7-EYFP; volumes were adjusted to result in equal levels of expression judged by expression of the EYFP reporter. All were infused at a rate of 0.16 μL/min and left in place for an additional 5 min (Figure 1A).

#### Spinal Cord Injury

The spinal cord was exposed with a laminectomy at T-9 and stabilized with vertebral clips. An NYU impactor was aligned with the exposed spinal cord and weight dropped from 25 cm to induce a severe contusion (48). Following surgeries, the incision site over the cord was closed in layers, animals were given 5 mL of lactated Ringers solution, and placed on a heating pad to recover thermoregulation.

### IVIS Imaging

Bioluminescence imaging was done under isoflurane anesthesia, with an IVIS Lumina LT (Perkin Elmer) where the CTZ was infused through the cannula and the animal was imaged for a time series with the exposure set at 5 min, f-stop at 1, with large binning.

### *In vivo* Electrophysiology Recordings

Acute recordings were performed under 1.2–1.5 g/kg urethane. Animals were secured in a Kopf spinal stereotax and a Hamilton syringe with a 25G beveled needle, loaded with CTZ was lowered into the lateral ventricle. A laminectomy was performed at the L1 vertebra and a 32 channel electrode array (A2x16, NeuroNexus) was lowered on one side of the cord to a depth of 2 mm. For acquisition, a Blackrock Microsystems CerePlex μ head stage and CerePlex Direct acquisition system were used. Recordings were filtered with a 250 Hz high pass fourth order Butterworth filter and single units were sorted using the Blackrock offline spike sorter or Blackrock online spike sorting software. After sorting, spikes were quantified using Neuroexplorer 5.

### Treatment

Water-soluble CTZ (Nanolight #3031) and CTZ solvent (Nanolight #3031C) were used throughout. For treatment, animals received 30 μL of CTZ (150 μg) or equivalent vehicle solvent, including ~7–10 μL cannula dead volume. Ventricular infusions were delivered at 4 μL/min every other day for 14 days beginning 1 day post injury. During infusions, animals were allowed to freely move in an open field.

### Behavioral Testing

Behavioral testing was done using the Basso, Beattie, and Bresnahan (BBB) rating scale for spinal cord injured rats, where rats are rated on a scale from 0 to 21, with 0 being completely paralyzed, 10 being the first point where weight bearing steps occur, and 21 having a perfect gait (49). All behavioral testing was done by two blinded observers. If behavior testing occurred on the same day as treatment, behavior testing was done prior to the CTZ mediated stimulation.

### Histology

At 5 weeks post injury, rats were given a lethal dose of Fatal Plus (Vortech Pharmaceuticals), and tissue was collected by transcardial perfusion with cold phosphate buffered saline (PBS) followed by 4% w/v paraformaldehyde solution in PBS. Spinal cords were extracted and incubated in the 4% paraformaldehyde solution at 4°C overnight. Prior to freezing, cords were acclimated to 30% sucrose in PBS w/v for 3 days at 4°C, then flash frozen and stored at −80°C. Thoracic and lumbar regions were embedded in M1 embedding matrix (Fisher Scientific), cryosectioned at 30 μm for thoracic and 50 μm for lumbar regions and mounted directly on positively charged slides for histological staining or fluorescent imaging.

For eriochrome cyanine (EC, Sigma) staining, thoracic sections mounted on slides were air dried, dehydrated, and defatted in graded ethanol solutions (50, 70, 90, 95, 100%, 3 min each) followed by xylene (10 min), rehydrated in graded ethanol solutions, then incubated in EC solution for 10 min (50). Slides were rinsed twice with water and differentiated in 0.5% ammonium hydroxide, then rinsed twice with water. Slides were dehydrated in graded ethanol solutions to xylene and cover slipped with Eukitt mounting media (Sigma). Slides were scanned with a Nikon Coolscan IV slide scanner. Spared white matter was quantified by tracings in ImageJ software by personnel blinded to condition.

### Gene Expression

Animals were deeply anesthetized with isoflurane, the spinal cord was dissected out, rinsed in cold PBS, and the lumbar enlargement placed into a tube and flash frozen in liquid nitrogen. Samples were stored at −80°C until processed. RNA was extracted using an All Prep kit (Qiagen) per the manufacturer's instructions. Quantitative RT-PCR (qPCR) was performed as previously described (51). Briefly, complementary DNA synthesis was performed using the High Capacity RNA-cDNA kit (Applied Biosystems). All samples were analyzed in triplicates using a StepOnePlus Real-Time PCR machine (Applied Biosystems) and Eva Green PCR Master Mix (MidSci) in a total volume of 20 μL. Gene expression was normalized to Glyceraldehyde 3-phosphate dehydrogenase (GAPDH). Results were analyzed using the double delta CT method and are expressed as fold expression of sham animals. Sham animals underwent the same surgery 1 as experimental animals but for surgery 2 only had a dorsal laminectomy.

### Statistics

All statistical tests were performed in SPSS Statistics 24 (IBM) or Prism 9. A two way repeated measures ANOVA was used for BBB with Bonferonni *post-hoc* test (49). For all other analysis, a one way ANOVA with Bonferonni *post-hoc* was used. Sample sizes were estimated using power analysis with G^*^power 3.1 (52).

## Data Availability Statement

The raw data supporting the conclusions of this article will be made available by the authors, without undue reservation.

## Ethics Statement

The animal study was reviewed and approved by Central Michigan University.

## Author Contributions

EP and UH designed the experiments and wrote the paper. EP initiated and conceptualized the study and carried out all experiments and analysis. ES performed animal care and treatment. AP performed animal care and surgery. LS performed animal care and treatment. JZ-P performed animal care, behavior, and treatment. AJP performed histology and analysis. AA performed data analysis. MP performed behavior. UH supervised all aspects of the work. All authors contributed to the article and approved the submitted version.

## Funding

This study was supported by the National Institutes of Health (U01NS099709), the National Science Foundation (DBI-1707352), the W.M. Keck Foundation, the Craig H. Neilsen Foundation, and the CMU Office of Research and Graduate Studies.

## Conflict of Interest

The authors declare that the research was conducted in the absence of any commercial or financial relationships that could be construed as a potential conflict of interest.

## Publisher's Note

All claims expressed in this article are solely those of the authors and do not necessarily represent those of their affiliated organizations, or those of the publisher, the editors and the reviewers. Any product that may be evaluated in this article, or claim that may be made by its manufacturer, is not guaranteed or endorsed by the publisher.
