# Supplementary material for: Restoring Function After Severe Spinal Cord Injury Through BioLuminescent-OptoGenetics
Source: Front Neurol. 2022 Jan 20;12:792643. doi: 10.3389/fneur.2021.792643 (PMC8811305; doi:10.3389/fneur.2021.792643)
Supplement: Supplementary file 1 [file Table_1.DOCX]

Supplementary figure SCI BL-OG


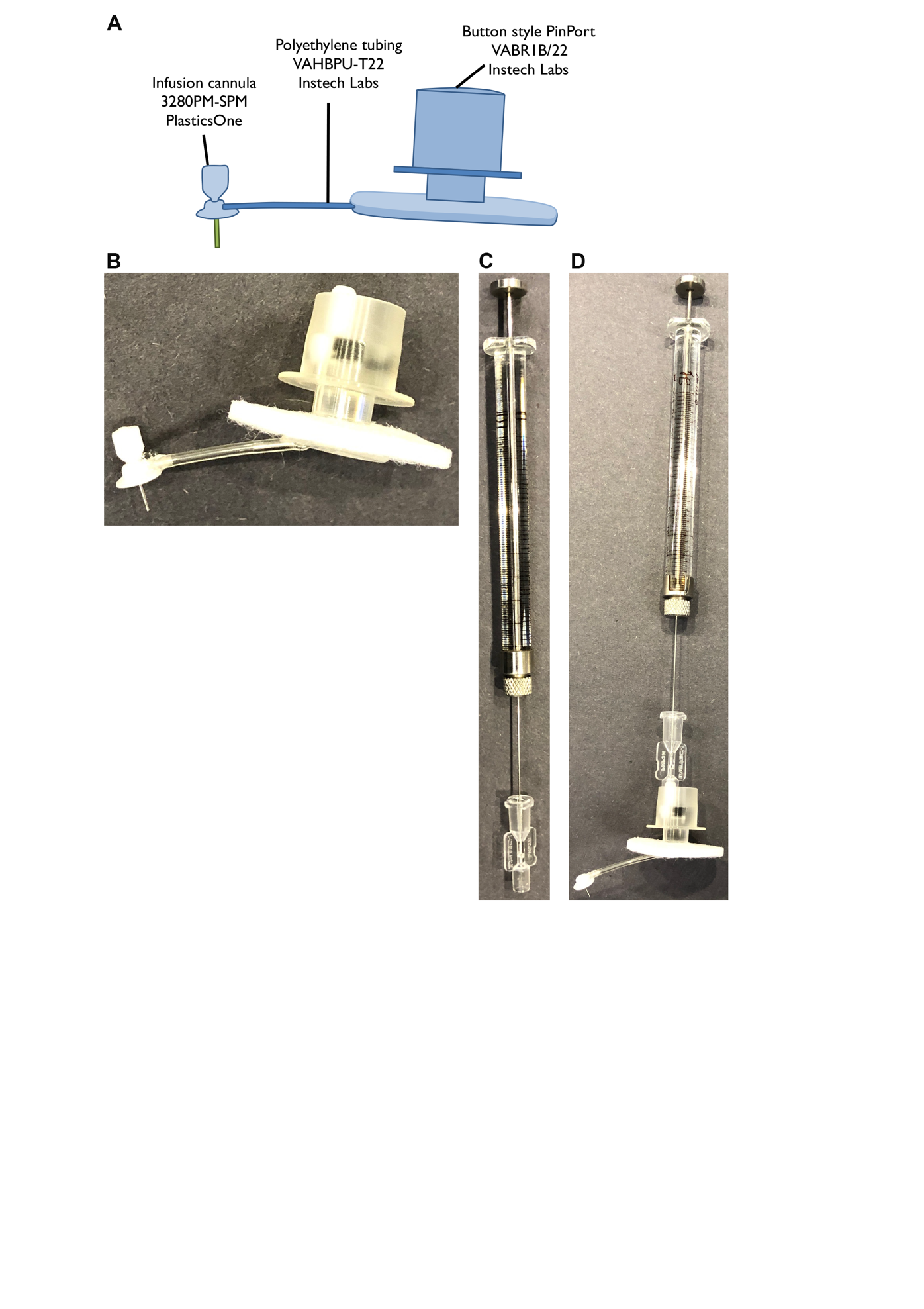


Figure S1. A. Schematic of the cannula used in this study consisting of an infusion cannula and PinPort button which are attached by polyethylene tubing and glued together. B. Picture of the assembled cannula. C. A 100µL Hamilton Gastight syringe with a modified 25G blunt needle. To assemble the needle, the metal insert of an Instech PinPort injector was removed, the hole in the center was expanded all the way through with a 25G beveled syringe needle, and the 25G blunt Hamilton needle is inserted to replace the original metal piece with the knurl positioned on the needle first. D. The assembled cannula with injector inserted as it would be for an infusion with this cannula. To our knowledge this is the first time these commercially available parts have been combined for ventricular cannulation. This approach allows researchers to acutely infuse substances into the brain without restraining the animal as would be necessary with other cannulation approaches.

Figure S2. Number of days until the return of bladder function. Those which received vehicle treatment did not regain bladder control as soon as those receiving CTZ, however this difference was not considered significantly different.

| Gene | Forward 5’-3’ | Reverse 5’-3’ |
| --- | --- | --- |
| GAP-43 | CAAGCTGAGGAGGAGAAAGAAGC | GGAGAGACAGGGTTCAGGTG |
| Map2 | CACTGGAAGAAGCCTCGAAGATG | TCCTTGTCTAAAGGCTCAGCG |
| NMDAR2d | ACCCTGACATGCACAGCTAC | TCCAGTTTCCCTGCCTTGAG |
| PSD-95 | AACACGGACACCCTAGAAGC | CAGACCTGAGTTACCCCTTTCC |
| BDNF | GAAGAGCTGCTGGATGAGGAC | TTCAGTTGGCCTTTTGATACC |
| VEGF | CAGAAAGCCCATGAAGTGGTG | GGGCTTCATCATTGCAGCAG |
| Caspace-3 | GAGCTTGGAACGCGAAGAAAAG | AGAGTCCATCGACTTGCTTCC |
| Bcl-2 | GGATAACGGAGGCTGGGATG | AGCAGCGTCTTCAGAGACAG |
| iNOS | ATCTTGGAGCGAGTTGTGGATTGTT | GGTAGTGATGTCCAGGAAGTAGGTGA |
| Arginase | TGTGGTAGCAGAGACCCAGAAGAAT | CAGCGGAGTGTTGATGTCAGTGT |

Table S1. List of primers used.
